# Editorial: New Insights Into the Transmission Dynamics and Control of Antimicrobial Resistance to Last-Resort Antibiotics

**DOI:** 10.3389/fmicb.2022.914978

**Published:** 2022-05-18

**Authors:** Zhuoren Ling, Shaolin Wang

**Affiliations:** ^1^Beijing Key Laboratory of Detection Technology for Animal-Derived Food Safety, College of Veterinary Medicine, China Agricultural University, Beijing, China; ^2^Department of Zoology, University of Oxford, Oxford, United Kingdom

**Keywords:** antibiotics, antimicrobial resistance, transmission, control, plasmid

Antimicrobial Resistance (AMR) is one of the most alarming public health issues around the world. Carbapenem, colistin, and tigecycline are currently the last-resort antibiotics for the treatment against multidrug resistant bacteria. This Research Topic integrates recent studies on the transmission dynamics and control strategies of AMR to these important drugs. It is about the mechanism of spread and the mitigation approaches of AMR.

## Dynamic Transmission of AMR

In this Research Topic collection, nearly half of the articles covered the critical role of plasmids in AMR dissemination.

On the one hand, plasmids carrying resistant genes acted as vehicles for horizontal gene transfer. Taking colistin resistance as an example, a well-documented gene, *mcr-1* has been frequently harbored on multiple transferable plasmids and spread worldwide. This gene encoded the MCR-1 enzyme that modified LPS and attenuated bacterial affinity with colistin (Liu et al., 2016). To better understand the interactome profile of MCR-1 with bacterial proteins, Li et al., using co-immunoprecipitation and mass spectrometry, found that MCR-1 affected protein biosynthesis by interacting with ribosomal proteins. Besides, they discovered that multidrug efflux pump AcrA-TolC was also involved in MCR-1-mediated colistin resistance. These data provided valuable information to a deeper understanding of MCR-1 function. Another study by Zhu et al. revealed that under colistin pressure, although *mcr-1* did not facilitate the evolution of high-level colistin resistance in *K. pneumoniae* and *E. coli*, it still improved the survival rates of the above species. Besides, high-level colistin resistance (HLCR) was more likely to emerge in *K. pneumoniae* than *E. coli* when exposed to colistin. This result partly explained why HLCR was more common in *K. pneumoniae* than *E. coli* in clinical sections.

Concerning tigecycline resistance, the plasmid-borne *tet*(X3) and *tet*(X4) were recently reported, alarming the possible horizontal spread of resistance (He et al., 2019; Zhang et al., 2020). This was further demonstrated by the research of Li et al. which identified one conjugative IncX1 plasmid habouring *tet*(X4) in *Escherichia fergusonii*, along with two chromosome-bearing *tet*(X6) in *Proteus cibarius*, from chicken feces. Apart from *tet*(X) and its variants, Xu et al. revealed that *tet*(A) mutation occurred under selective pressure could also lead to tigecycline resistance in *K. pneumoniae*. In their study, induction experiment was performed in which 71.4% (20/28) of *tet*(A)-carrying tigecycline resistance *K. pneumoniae* developed *tet*(A) mutations. And twelve (12/20) of them successfully transferred their *tet*(A)-mutant plasmids to *E. coli* EC600 by conjugation and led to an elevated level of tigecycline MIC in recipients (Xu et al.). These data indicated that mutations of *tet*(A) in conjugative plasmids may contribute to the tigecycline resistance in *K. pneumoniae* and *E. coli*.

To better understand the evolution of plasmids related to carbapenem resistance, one study by Liu et al. analyzed 84 non-duplicate IncX3 plasmids reported across China from 2011 to 2021. And all these plasmids harbored carbapenemase genes, among which 81 were *bla*_NDM_ carrying and 3 were *bla*_OXA−181_-carrying. In those NDM-positive plasmids, the NDM-5-positive ones were dominant and recovered from diverse sources, including clinical specimens, animals, environment and retail food, suggesting the wide spread of NDM-5-producing isolates through the transfer of IncX3 plasmids. However, NDM-1-positive IncX3 plasmids were mainly identified in the hospital section, which was disseminated at a limited level compared with NDM-5. The genetic context of *bla*_NDM_ on IncX3 plasmid could be classified into five subtypes, two of which have been identified in *Enterobacter cloacae* chromosome and IncF & IncA/C plasmids, respectively. This indicated that the *bla*_NDM_ gene environment could be transmitted by different plasmids and bacterial species. The other two studies reported KPC-2-carrying plasmids in China from *P. aeruginosa* (pP33-2, non-conjugative) and *E. coli* (pEC2341-KPC conjugative; pEC2547-KPC non-conjugative), respectively (Cai et al.; Wu et al.). In all the three plasmids, KPC-2 was bracketed by Insertion Sequence (IS), suggesting the potential role of IS in promoting the spread of KPC-2 among different plasmids.

On the other hand, plasmid could also act as a “lending library,” integrating its sequence into bacterial chromosome and promoting the vertical transmission of AMR. Zhang et al. found that, to cause colistin-resistance in *K. pneumoniae* under drug pressure, IS*Kpn72* on a plasmid could be inserted into *mgrB* gene with a higher efficiency than the IS*Kpn72* from chromosome. More importantly, this plasmid was conjugative, providing a clue about the widespread of *mgrB* inactivation among *K. pneumoniae* in hospitals (Cannatelli et al., 2014). In another research, Wang et al. reported the first detection of clinical *E. coli* (ST131) with a *bla*_KPC−2_ in chromosome. Interestingly, sequence analysis showed that the chromosomal *bla*_KPC−2_ was carried in a 24 kb IS with high similarity to the *bla*_KPC−2_-habouring plasmids in *P. mirabilis* (Hua et al., 2020). This result suggested that the *bla*_KPC−2_ was probably transferred from the *P. mirabilis* plasmid to *E. coli* chromosome through IS element. Once incorporated into chromosome, AMR mechanism would largely be maintained in bacterial replication and worth clinical attention.

In recent years, the progress in whole genome sequencing (WGS) made it easier to study the AMR transmission in a deeper level. Using this technique, two studies investigated the evolution of AMR isolates. Gostev et al. uncovered the existence of three international lineage of linezolid-resistant *S. epidermidis*. And Zhao et al. sequenced 99 Carbapenem-Resistant *Klebsiella pneumoniae* (CRKP) from the intensive care unit (ICU) and provided a glimpse of intraspecies replacement in the hospital. According to phylogenetic analysis, both ST4496-KL47 and ST11-KL64 were likely to originated from ST11-KL47. However, ST4496-KL47 was less competitive than ST11-KL64 and disappeared after 6 months. While ST11-KL64 increased virulence by capsule biosynthesis locus recombination and acquired the potential to be the dominant CRKP in hospital. In addition, the serotype was found to be associated with antimicrobial resistance in this Research Topic. The study by Luo et al. indicated that, for clinical Salmonella isolates in China, colistin resistance mainly distributed in *S. Enteritidis* (83.9%, 125/149) and *S. Typhimurium* (15.3%, 9/59), while the resistant rate of the former was significantly higher than that of the latter.

## Control of Antimicrobial Resistance

To put AMR under control, various strategies need to be executed, including constant surveillance of drug resistance, fast diagnosis of AMR bacteria infections and development of novel therapy.

Firstly, the spread of drug resistance needs to be put under surveillance. The work by Chang et al. revealed the epidemiology and change of antimicrobial resistance rates in children patients with non-typhoidal Salmonella infection from 2012 to 2019. And the report by Paveenkittiporn et al. provided comprehensive information to the prevalence of *mcr*-positive Carbapenem-Resistant Enterobacteriaceae (CRE) among patients in Thailand from 2016 to 2019. Another study by Ma et al. investigated AMR in clinical *Ureaplasma* spp. and addressed the lack of data in this field in China. In their research, the most active antibiotics were azithromycin, josamycin, and clarithromycin. These data provided updates of the prevalence of AMR in hospitals and facilitated clinicians for the rational use of antibiotics.

Secondly, reliable methods for rapid detection of AMR are of great importance in helping clinicians selecting effective antibiotics precisely, optimizing the treatment and preventing the aggravation of infection by Multi-Drug Resistant (MDR) microbes. To compare the currently available methods for fast carbapenemase detection, Han et al. investigated the performance of three lateral flow chromatographic assays (NG-test Carba 5, RESIST-5 O.O.K.N.V., and IMP K-SeT) in determining carbapenemases among CRE. As a result, the NG-test Carba 5 detected all of its targeted carbapenemases (KPC, NDM, VIM, IMP and OXA-48-like) in 15 min with both sensitivity and specificity at 100%. The RESIST-5 O.O.K.N.V. detected KPC, NDM, VIM and OXA-48-like with the sensitivity and specificity at 99.4 and 100%, respectively. The IMP K-SeT, as a complementary test of RESIST-5 O.O.K.N.V for IMP determination, detected all six IMP producers in the research. This result showed that all the above detection methods could simplify the complex workflow for carbapenemases identification for infection control purposes and epidemiological surveillance. For upgrading detection approaches, Jia et al. developed an AI-Blue-Carba test, an improved version of Blue-Carba, to determine carbapenemase-producing Gram-negative bacteria. In this research, “deep learning” was innovatively combined with traditional Blue-Carba, which allowed elevated efficiency for the detection of carbapenemases (within 15 min) and was user-friendly. Through using AI-Blue-Carba, the author planned to create a co-networking platform with a hospital which would promisingly facilitate the monitoring of carbapenemases and infection treatment in clinical sections. Another study explored a method for fast detection of *Staphylococcus aureus* from patients with diabetic foot infections (Chen et al.). In this method, loop-mediated isothermal amplification (LAMP) and clustered regularly interspaced short palindromic repeats (CRISPR) techniques were coupled. Through targeting *nuc* and *mecA*, the CRISPR-LAMP assay successfully identified *S. aureus* strains and distinguished methicillin-resistant *S. aureus* (MRSA) from methicillin-resistant *S. aureus* (MSSA) in clinical samples. Due to its visualized fluorescent result with easy interpretation criteria, this method could help the point-of-care diagnosis against MRSA. More tools are worth being investigated for fast and precise diagnosis of AMR, like aptamers and CRISPR-Cas suggested in the review by Pereira et al. The former (aptamers) could recognize bacteria through binding to cell surface receptors, antigens or unknown targets, while the latter (CRISPR-Cas) could recognize and cleave the targeted bacterial nucleic acid. Their application for diagnostics has been demonstrated in different studies to offer shorter turnaround time comparing with the conventional AMR phenotypic tests (Pereira et al.), highlighting the potential of these techniques to be refined and used as guidance to clinical antimicrobial prescriptions.

Thirdly, the development of innovative therapies are urgently needed for anti-infective purpose. One straight forward way is selecting new antimicrobial candidates. A novel antibiotic, named lefamulin, has been approved by the U.S. Food and Drug Administration in 2019 against community-acquired bacterial pneumonia (CABP). And Wu et al. evaluated its *in vitro* antibiogram by testing the minimum inhibitory concentrations (MICs) of lefamulin against 634 clinical respiratory pathogens across China. They demonstrated that lefamulin had excellent antimicrobial activity with a broad-spectrum coverage of respiratory pathogens, including MRSA/MSSA, methicillin-resistant/sensitive *S. epidermidis* (MRSE/MSSE), *Streptococcus. pneumoniae*, β-hemolytic *Streptococcus, Haemophilus influenzae, H. parainfluenzae, Morazella catarrhalis*, and *Mycoplasma. pneumoniae*. The *in vitro* activity of lefamulin supported the use of this drug as an alternative treatment option for CABP in China. Apart from new drugs, a reasonable combination of currently available antibiotics is another choice. Chen et al. found that CRKP developed resistance in tigecycline-induced descendants, while in the meantime, also presented stable hypersensitivities to other antibiotics, especially aminoglycosides, showing significantly lower MICs. Further genetic analysis suggested that the loss of AMR plasmids may play a role in this phenomenon. These new findings supported the combination therapy of tigecycline and aminoglycosides against CRKP infections.

To summarize, this Research Topic brought together a number of articles addressing the dynamics of AMR transmission and novel strategies of controlling AMR bacteria infection. Based on these new insights, plasmids were re-emphasized as a critical driving force in the spread and evolution of bacterial drug resistance, not only as mobile gene carriers, but also gene lenders to chromosomes. To overcome the problem of AMR, medical progress is being made in the fields of novel therapies and fast infection diagnosis which promotes the rational use of antimicrobials in the future.

## Author Contributions

All authors listed have made a substantial, direct, and intellectual contribution to the work and approved it for publication.

## Funding

This work was supported by the Laboratory of Lingnan Modern Agriculture Project (NT2021006) and China Agriculture Research System (CARS-36).

## Conflict of Interest

The authors declare that the research was conducted in the absence of any commercial or financial relationships that could be construed as a potential conflict of interest.

## Publisher's Note

All claims expressed in this article are solely those of the authors and do not necessarily represent those of their affiliated organizations, or those of the publisher, the editors and the reviewers. Any product that may be evaluated in this article, or claim that may be made by its manufacturer, is not guaranteed or endorsed by the publisher.
